# Mindfulness-based cognitive therapy improves insomnia symptoms in individuals with recurrent depression: secondary analyses from a randomized controlled trial

**DOI:** 10.3389/fpsyt.2023.1231040

**Published:** 2024-01-19

**Authors:** Linn Nyjordet Evanger, Elisabeth Flo-Groeneboom, Lin Sørensen, Elisabeth Schanche

**Affiliations:** ^1^Department of Global Public Health and Primary Care, Faculty of Medicine, University of Bergen, Bergen, Norway; ^2^Department of Clinical Psychology, Faculty of Psychology, University of Bergen, Bergen, Norway; ^3^Department of Biological and Medical Psychology, Faculty of Psychology, University of Bergen, Bergen, Norway

**Keywords:** MBCT, mindfulness, psychotherapy, recurrent depression, insomnia, sleep

## Abstract

**Background:**

Embedded within a randomized efficacy trial, the present study aimed to investigate whether mindfulness-based cognitive therapy (MBCT) for recurrent depression improved symptoms of insomnia.

**Methods:**

Sixty-eight remitted participants with at least three prior episodes of depression were randomized to 8 weeks of MBCT (*n* = 33) or a waitlist control condition (*n* = 35). The Bergen Insomnia Scale was used to screen for insomnia symptoms before and after the intervention. The analyses were conducted using one-way between-groups analyses of covariance.

**Results:**

Twenty-five MBCT participants and 30 waitlist controls completed the study (74.5% females; mean age 40.7 ± 12.9 years). At baseline, 83.6% screened positive for the insomnia diagnosis. Following the intervention and after controlling for corresponding insomnia parameters at baseline, MBCT completers reported significantly less severe insomnia symptoms (*p* = 0.017), as well as less problems with prolonged sleep onset (*p* = 0.047) and nocturnal awakenings (*p* = 0.023), relative to controls. No group differences were found on early morning awakening, daytime impairment, or dissatisfaction with sleep.

**Conclusion:**

The results suggest that MBCT improves certain insomnia symptoms. However, additional sleep-specific interventions may be needed to further improve this population’s sleep.

**Clinical Trial Registration**: ISRCTN, ISRCTN18001392, registered 22/11/2018. URL: 10.1186/ISRCTN18001392.

## 1 Introduction

Recurrent depressive disorder, broadly defined as an episode of major depression disorder (MDD) along with a history of at least one previous depressive episode, is a highly prevalent, chronic, and often disabling mental health disorder (1–6). Depression has been associated with numerous adverse outcomes, including increased risk of medical and psychiatric conditions (7, 8) and increased societal costs (9). It is therefore important, both for the suffering individual and in societal terms, to reduce the rate of recurring MDD cases (10).

It is well-established that many patients with depression also suffer from insomnia, and that the two disorders are bidirectionally related (11–13). Insomnia is a subjective sleep disorder characterized by problems initiating or maintaining sleep, or insufficient sleep quality (1, 11). To qualify for the chronic insomnia diagnosis, the International Classification of Sleep Disorders requires that at least one of the above sleep symptoms, along with daytime impairments (e.g., cognitive, social or occupational) and/or sleep-related worry, must have been present for at least three times per week for 3 months (11). Insomnia co-occurring with depression was previously assumed to be secondary to (i.e., a symptom of) the depressed state (12), and has consequently received little attention in the treatment context of MDD (10, 13). However, the past decades has recognized insomnia as a separate psychological disorder that in many cases develops prior to depression (14) and, once established, increases the risk of depressive relapse and recurrence (10, 15–17). Such findings, along with the tendency of insomnia to persist if left untreated (18), indicate that treating co-existing insomnia may be essential to ensure long-term recovery from depression (10, 19, 20).

Mindfulness-based cognitive therapy (MBCT) (21) is effective in reducing the risk of depressive relapse and recurrence (22–24), and is therefore recommended by the UK National Institute of Health and Clinical Excellence for the treatment of recurrent MDD (25). However, despite of evidence that insomnia may predict relapse and recurrence of MDD, few previous studies have investigated MBCT’s potential effects on insomnia (26, 27). Consequently, it is unclear whether recurrently depressed individuals with co-existing insomnia symptoms should be offered additional sleep-directed treatment interventions, in addition to being treated with MBCT.

A growing body of research suggests that mindfulness-based interventions (MBIs) may improve symptoms of insomnia (26–29). Accordingly, MBIs have been raised as alternative treatment options to cognitive behavioral therapy for insomnia (CBT-I), as some individuals do not respond adequately to this otherwise first-choice treatment for insomnia (29–32). Potential mechanisms underpinning MBIs’ effects on insomnia are currently less understood (31). However, preliminary evidence suggest that these interventions may improve several of the psychological and behavioral processes assumed to perpetuate the disorder (31). For example, and of particular relevance for insomnia comorbid with recurrent MDD, the chronicity of both depression and insomnia appear to be related to tendencies toward ruminative thinking (i.e., repetitive thinking about potential causes, ongoing experience, and potential consequences of one’s current symptoms and distress) (31–34). As MBCT has been found to improve ruminative thinking as well as several other psychological and behavioral processes characteristic for insomnia, it is reasonable to assume that MBCT may also improve insomnia, in addition to reducing the risk of depressive relapse and recurrence (31).

To the best of our knowledge, only one previous randomized controlled trial (RCT) has explored the potential effects of MBCT on sleep complaints among recurrently depressed individuals (35–37). This trial resulted in two separate papers, both by Britton and colleagues (35, 36), with somewhat contradicting findings. Their 2010 paper, based on 26 antidepressant-free participants, indicated that neither polysomnographic- nor sleep-diary based sleep improved following MBCT, relative to a waitlist control condition. Instead, MBCT was associated with marginally increases in polysomnographic-bases awakenings and less slow-wave sleep. By contrast, their 2012 paper, based on 23 antidepressant users, suggested that MBCT was associated with reduced total wake time and improved sleep efficiency relative to the waitlist control condition. The contradicting results in the studies by Britton and colleagues, and the limited research base on potential effects of MBCT on insomnia symptoms, suggest that more research on this area is needed.

The present study aimed to explore whether MBCT for recurrent depression improved symptoms of insomnia among remitted individuals with at least three prior depressive episodes. It was also of interest to explore the prevalence rate of insomnia in this subpopulation of depression-afflicted individuals. Based on previous findings that MBCT has been shown effective in improving processes underlying the symptomatology of both recurrent MDD and insomnia (e.g., rumination) (33, 38, 39), we predicted that MBCT would alleviate symptoms of insomnia. Also, and based on the comorbid relationship between depression and insomnia (40, 41), it was predicted that a relatively high proportion of the sample would qualify for the insomnia diagnosis at baseline.

## 2 Materials and methods

### 2.1 Trial design

The present study is part of a larger randomized wait-list controlled trial (Trial no ISRCTN18001392, registered 22/11/2018) (6) investigating the effects of MBCT on risk and protective factors of MBCT. Primary outcomes of this trial have been published with a detailed methods description (6), showing that MBCT effectively improved these patients’ emotion regulation strategies, depressive symptoms, and self-compassion- and mindfulness skills, while temporarily reducing their tendencies toward rumination and emotional reactivity to stress. The current study uses secondary analyses of data from baseline- and post-intervention assessments of insomnia, conducted before and after the eight-week MBCT intervention. The dependent variables were: (i) The overall severity of insomnia symptom at post-intervention, and (ii) levels of specific insomnia symptoms (sleep onset latency, wake after sleep onset, early morning awakening, non-restorative sleep, daytime impairment, and sleep-related dissatisfaction) following the intervention. This design would allow for detection of changes over time (baseline to post-assessments) while statistically controlling for within-subjects effects.

In the main trial of which the present was based (6), the minimum clinically meaningful difference between the treatments we sought to detect in Beck’s Depression Inventory (BDI) was 7. We also accepted a probability of Type 1 error of 5% with 80% power. Based on a previous study by Kuyken et al. (24) reporting an attrition rate of approximately 15% in their MBCT group, we expected an attrition rate below 20%. Based on this, approximately 30 participants were planned recruited to each treatment condition. A statistical power analysis (performed using G*power) indicated that this sample size would provide acceptable power.

### 2.2 Setting

Participants were recruited between May 2016 and August 2017, in three separate groups. All pre- and post-intervention assessments were administered at the University of Bergen, Norway, between January and October 2017. The pre- and post-intervention assessments were conducted within a time range of 2 weeks prior to and following the intervention period, respectively.

### 2.3 Participants

#### 2.3.1 Eligibility criteria

Subjects were eligible for participation if they: (1) Were at least 18 years old; (2) met the criteria for recurrent depressive disorder in either partial or full remission; and (3) had experienced at least three prior depressive episodes. Continuation of antidepressant medication use was allowed if no changes in dosage were made between the baseline and post-intervention assessment phases.

#### 2.3.2 Exclusion criteria

Potential participants were excluded if they: (1) Had any severe comorbid psychological disorder (schizophrenia, any lifetime history of psychosis, or bipolar disorder); (2) fulfilled the criteria for another treatment-needing disorder (borderline personality disorder, a serious eating disorder, post-traumatic stress disorder, or severe obsessive-compulsive disorder); (3) had an ongoing substance use disorder; (4) had a neurological or hormonal disease; (5) had a lifetime history of severe cardiovascular disease; (6) contemporarily attended another form of psychotherapy at least two times per month; (7) had received any type of mindfulness-based treatment during the past 24 months; and/or (8) were currently pregnant or breastfeeding.

### 2.4 Sampling procedures

Participants were recruited through general practitioners and psychiatric outpatient clinics in Bergen, Norway, via newspapers advertisements, and through mental health-related groups on the social media channel Facebook. Subjects who were interested in participation were asked to contact the research team through e-mail or phone. A brief screening based on the inclusion criteria was thereafter administered via phone. Among these, 82 potential participants were further assessed for eligibility using a Norwegian version of the Mini-International Neuropsychiatric Interview (M.I.N.I.) (42, 43). The M.I.N.I. is a semi-structured, diagnostic interview based on the DSM-IV and ICD-10 criteria for the most common axis 1 psychological disorders, including major depressive disorder (first episode or recurrent), substance use disorder, psychotic disorders, and antisocial personality disorder. Levels of depressive symptoms were assessed using the Hamilton Depression Rating Scale (HAM-D) (44). In addition, the borderline personality disorder module of the Structured Clinical Interview for DSM-IV AXIS II personality disorders (SCID-II) (45) was carried out to exclude potential participants with borderline personality disorder. M.I.N.I. and SCID-II were administered by clinical psychologists with extensive clinical training and experience. Demographics, as well as participants’ estimated age of the first depressive episode and time in remission, were also collected during the assessment interviews, based on retrospective recall of the participants.

Following baseline assessments, participants were randomly assigned to either the MBCT condition or the waitlist control condition. To ensure that both conditions were equal in terms of gender, the randomization process was stratified by gender. The randomization process was carried out by a colleague not involved in the project, using the random number generation function of Microsoft Excel (Microsoft Inc., Redmond, WA). Four to eight weeks following the clinical assessments, participants were re-invited to the laboratory to complete self-report questionnaires, including symptoms of insomnia and depression.

### 2.5 Interventions

#### 2.5.1 MBCT

Participants randomized to the MBCT condition received 8 weeks of MBCT, in line with the original manual by Segal and colleagues (21). The participants received the intervention in groups of eight to 14 participants at the University of Bergen, in three separate groups. In each weekly session, the participants were introduced to a theme relevant for understanding how habitual modes of thinking, feeling, and behaving may contribute to depressive relapse. The participants were also introduced to various practices that aimed to facilitate being mindful of, and relating to, bodily sensations, emotions, and thoughts in a more flexible manner. Participants were encouraged to relate mindfully not only to depression-related inner contents, but to any sort of psychological distress. Additionally, the participants were invited to conduct homework assignments between each session for approximately 45 min per day, 6 days a week. Materials necessary for the home assignments were translated into Norwegian and available for the participants through a webpage. The intervention was instructed in pairs of two clinical psychologists. In total, three therapists with extensive training in MBCT were involved.

#### 2.5.2 Control condition

The control condition was a waitlist control (WLC) condition in which participants received no treatment during the 8 weeks between the baseline and post-intervention assessments. WLC participants were offered the MBCT program after the pre- and post-assessments had been conducted.

### 2.6 Baseline and outcome assessments

#### 2.6.1 Beck depression inventory-II

A Norwegian validated translation of the Beck depression inventory-II (BDI-II) (46, 47) was used to assess levels of depression during the baseline- and post-intervention assessments. BDI-II is a 21-item self-report inventory assessing emotional, cognitive, physiological, and behavioral symptoms of depression. A total quantitative score of 0–13 indicates no or minimal depression; 14–19 indicates mild depression; 20–28 indicates moderate depression; and 29–63 indicates severe depression. The scale shows good psychometric properties (48). BDI-II had a high internal consistency in the current study (Cronbach’s α = 0.88).

#### 2.6.2 Mini-international neuropsychiatric interview

The mini-international neuropsychiatric interview (M.I.N.I) was used both to assess for eligibility according to the inclusion and exclusion criteria, including whether participants qualified for the recurrent subtype of Major depressive disorder. During the initial interviews, participants were also asked about time in remission and how many depressive episodes they had experienced.

#### 2.6.3 Bergen insomnia scale

The Bergen Insomnia Scale (BIS) (49) was used to assess insomnia during the pre- and post-intervention assessments. BIS is a six-item self-report measure based on the Diagnostic and Statistical Manual of Mental Disorders, 4th edition (DSM-IV, 1994) criteria for insomnia (12, 49). The scale asks how many days per week, on average, each of six insomnia symptoms have been present during the past month along an 8-point scale, ranging from 0 to 7 days a week. The first item targets how many days per week (0–7), on average, the respondent has experienced sleep onset latency (SOL, defined as sleep onset >30 min). Item 2 targets problematic wake after sleep onset (WASO; defined as awakenings >30 min after the sleep first set in and before one’s final awakening time). Item 3 targets early morning awakening (EMA; defined as final awakening more than 30 min before one’s desired wake-up time). Item 4 asks how many days (0–7) the respondent’s sleep has been experienced as nonrestorative. Item 5 asks how many days per week (0–7) the respondent has experienced daytime sleepiness interfering with private, educational, or occupational activities. Finally, item 6 asks how many days per week (0–7) the respondent has been dissatisfied with her/his sleep.

The respondent is qualified for the insomnia diagnosis if at least one of items 1–4 and at least one of items 5 and 6 have been present for at least 3 days weekly during the past month. Additionally, a total score is obtained by adding up the value of each item, resulting in a total value ranging from 0 to 42 (referred to as “BIS Total” in the following sections), of which higher score indicates more severe overall burden of insomnia symptoms. The scale shows good psychometric properties (49). BIS had a Cronbach’s α of 0.69 in the present study.

For between-group comparisons (MBCT versus WLC), specific insomnia symptom (BIS items 1–6) as well as BIS Total were measured continuously along their respective 0–7 and 0–42 BIS scales. For exploration of the baseline insomnia rate, the full insomnia diagnosis was defined as met if participants fulfilled the BIS criteria for the insomnia diagnosis. Specific insomnia symptoms were defined as met if they were rated as present for an average of at least 3 days a week during the past month.

### 2.7 Ethics statement

The study followed the Helsinki declaration guidelines and the Vancouver rules. All pre-screening assessments were conducted by experienced clinical psychologists. No economic compensation was given for participation. However, WLC participants were offered MBCT after the post-intervention assessments were completed, and participation was free of charge. All participants provided written consent and were told they could withdraw from the study at any time. The study was approved by the Norwegian Regional Committee for Medical and Health Research Ethics of of South East Norway, University of Oslo, Norway (study number 2016/388).

### 2.8 Statistical methods

All analyses were conducted using IBM SPSS Statistics, version 25. Independent samples *t*-tests, chi-square tests for independence, and Fisher’s Exact Probability Test were performed to ensure that the treatment conditions were equal in terms of age, gender, and depression and insomnia levels, prior to the intervention. Additionally, Fisher’s Exact Probability Tests were performed to check whether the conditions differed in terms of comorbid psychiatric conditions, based on the initial M.I.N.I. screenings. Bivariate correlation analyses were conducted to check whether any of the included insomnia parameters correlated with time in remission or any of the other included insomnia variables. Preliminary checks were performed to test for violation of the assumptions of normality, linearity, homogeneity of variances, and homogeneity of regression slopes for each of the included insomnia parameters. The sample was checked for outliers, and missing values were replaced (for details, see Schanche et al., 2020) (6). All alpha levels were set at 0.05, two-tailed.

One-way between-groups analyses of covariance (ANCOVAs) were performed to compare the effects of MBCT and WLC on post-intervention levels of insomnia (BIS Total as well as BIS items 1–6) while adjusting for baseline levels of corresponding insomnia parameters.

## 3 Results

### 3.1 Participant flow

**Figure 1:**
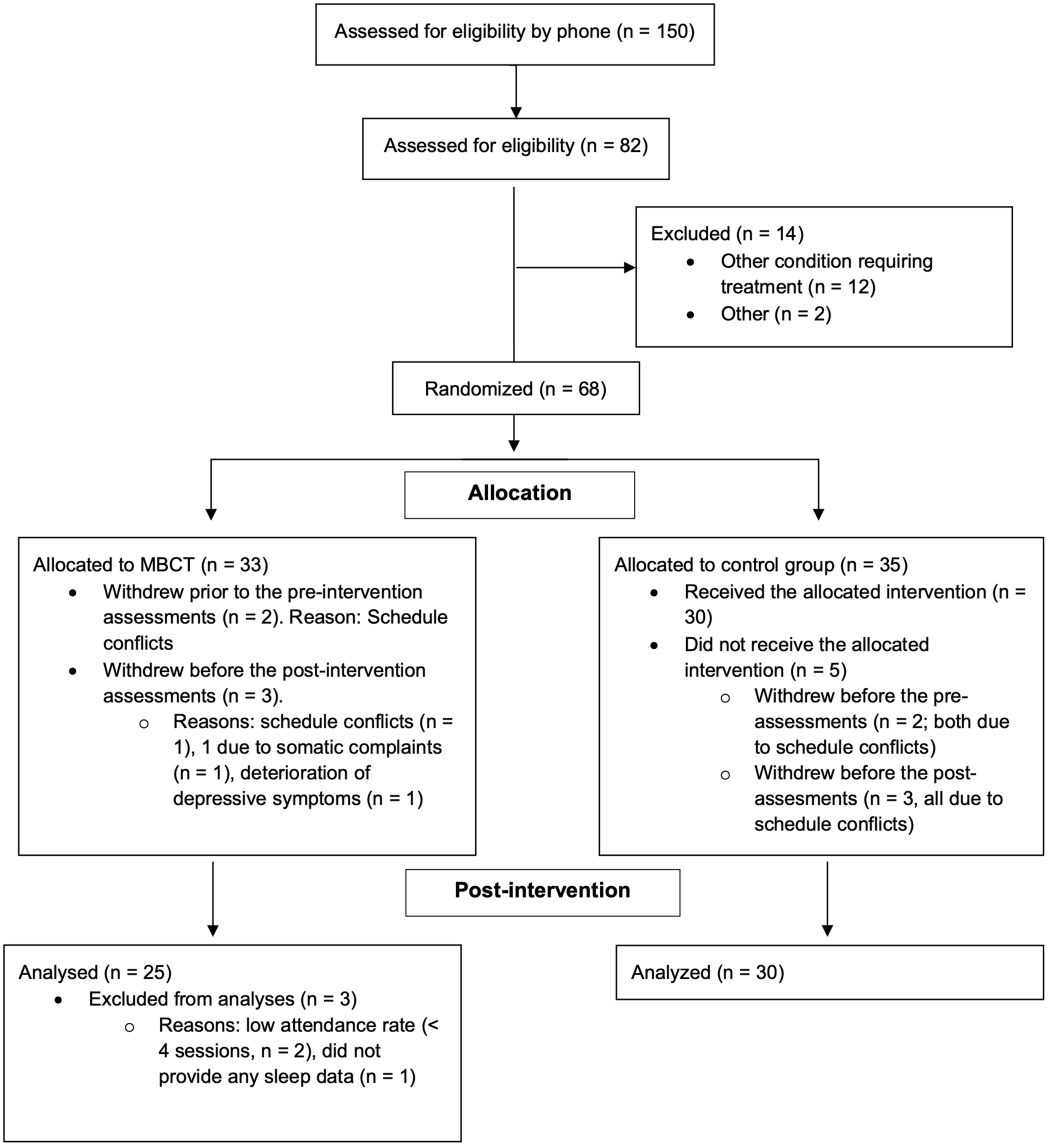
Flow over participants.

Sixty-eight participants were rated as eligible for participation and randomly allocated to either MBCT (*n* = 33) or WLC (*n* = 35). Twenty-eight participants allocated to the MBCT condition received the intervention and attended the baseline- and post-intervention assessments. Two participants randomized to the MBCT condition were excluded from further analyses due to low attendance rate (less than four sessions) during the MBCT course. One further MBCT participant was excluded as this participant did not respond to any of the BIS items neither during the baseline- or post-intervention assessments. Among the 35 participants allocated to WLC, five withdrew due to schedule conflicts; two during the baseline assessments and another three during the post-assessment phase. Thus, the final analyzed sample comprised 55 participants (mean age = 40.7 years; SD = 12.9; 74.5% females); 30 WLC and 25 MBCT participants. All participants of the final analyzed sample responded to all BIS items during both pre- and post-intervention assessments. For detailed flow of participants, please see Figure 1.

### 3.2 Baseline analyses

For baseline distribution of the included insomnia parameters, please see Table 1. No differences in terms of age, gender, the rate of participants screening positive for the insomnia diagnosis, comorbid psychiatric conditions according to the M.I.N.I. screenings, BIS Total or BDI-II scores were found between the treatment conditions at baseline (all *p-*values >0.05). Most of the participants reported being married/cohabiting and fulltime working, and the majority reported having education above high school and/or vocational qualification. Furthermore, less than a third of the sample reported using some sort of antidepressant medication. For further details on participant characteristics, please see Schanche and colleagues, 2020 (6).

**Table 1:** Baseline descriptive statistics for each treatment condition (MBCT or WLC) and the overall sample among study completers.

	**MBCT (*n* = 25)**	**WLC (*n* = 30)**	**Overall sample (*N* = 55)**	***p* value**
Mean age, years (SD)	42.1 (12.9)	39.6 (13.1)	40.7 (12.9)	0.471^a^
Women (%)	17 (68.0%)	24 (80.0%)	41 (74.5%)	0.480^b^
Overall insomnia severity^1^, *M* (SD)	20.6 (8.1)	19 (6.8)	19.7 (7.4)	0.429^a^
**Specific insomnia symptoms^2^, M (SD)**				
Sleep onset latency	2.8 (2.0)	3.6 (2.3)	3.2 (2.2)	0.142^a^
Wake after sleep onset	2.4 (1.9)	1.9 (1.6)	2.1 (1.8)	0.374^a^
Early morning awakening	2.3 (2.2)	2.4 (1.9)	2.4 (2.1)	0.786^a^
Nonrestorative sleep	5.0 (1.8)	4.2 (1.8)	4.6 (1.9)	0.096^a^
Daytime impairments	3.6 (2.1)	2.5 (2.0)	3.0 (2.1)	0.053^a^
Dissatisfaction with sleep	4.6 (1.9)	4.3 (1.7)	4.4 (1.8)	0.595^a^
Insomnia by diagnosis, *N* (%)	21 (84.0%)	25 (83.3%)	46 (83.6%)	1.000^c^
**Comorbid psychiatric conditions (M.I.N.I.)**				
Panic Disorder, ongoing	3 (12.0%)	0 (0.0%)	3 (5.5%)	0.088^c^
Panic Disorder, lifetime	3 (12.0%)	8 (26.7%)	11 (20.0%)	0.310^c^
Agoraphobia	5 (20.0%)	3 (10.0%)	8 (14.5%)	0.446^c^
Social anxiety, generalized	1 (4.0%)	2 (6.7%)	3 (5.5%)	1.000^c^
Social anxiety, nongeneralized	1 (4.0%)	1 (3.3%)	2 (3.6%)	1.000^c^
Generalized anxiety disorder	5 (20.0)	6 (20.0%)	11 (20.0%)	1.000^b^

MBCT, mindfulness-based cognitive therapy; WLC, waitlist control; M, mean; SD, standard deviation; N, number of participants; M.I.N.I., based on Mini-international neuropsychiatric interview.^1^Based on the composite score of the Bergen Insomnia Scale; ^2^Mean Bergen Insomnia subscale scores = mean number of days per week experiencing the given symptom.^a^based on independent samples *t*-test; ^b^based on chi-square test for independence (with Yates’ continuity correction); ^c^based on Fisher’s exact test.

Descriptive analyses showed that 46 of the 55 participants (83.6% of the overall sample) screened positive for the insomnia diagnosis at baseline. The most prevalent insomnia symptom was nonrestorative sleep, as reported by 83.6% of the sample, followed by prolonged sleep onset (reported by 60.0% of the sample).

### 3.3 Treatment effect

Estimated marginal means and standard errors of all ANCOVA analyses are presented in Table 2. After the intervention, MBCT participants reported less severe overall insomnia symptoms, relative to control participants (*p* = 0.017). In terms of specific insomnia symptoms, MBCT participants reported less problems with SOL (*p* = 0.047) and WASO (*p* = 0.023) compared to control participants. However, the groups did not report differences on EMA (*p* = 0.169) or nonrestorative sleep (*p* = 0.429) at post-intervention after controlling for corresponding sleep parameters at baseline. Likewise, no group differences were found on sleep-related daytime functioning (*p* = 0.190) or dissatisfaction with sleep (*p* = 0.067, Table 2) following intervention when adjusting for corresponding baseline variables.

**Table 2:** Results from one-way between-groups analyses of covariance (ANCOVAs) for each treatment condition (MBCT or waitlist control), showing the distribution of insomnia symptoms based on estimated marginal means and standard errors.

	**MBCT (*n* = 25)**		**WLC (*n* = 30)**		**ANCOVA (*N* = 55)**		
	***M***	**SE**	***M***	**SE**	***F***	***p***	**SE**
**Overall insomnia severity**							
BIS Total^1^	14.81	1.57	20.06	1.43	6.071	0.017*	0.105
**BIS subscales**^2^							
Sleep onset latency	2.38	0.32	3.25	0.29	4.122	0.047*	0.073
Wake after sleep onset	1.47	0.37	2.64	0.33	5.517	0.023*	0.096
Early morning awakening	1.31	0.36	1.98	0.32	1.948	0.169	0.036
Nonrestorative sleep	3.95	0.44	4.44	0.40	0.634	0.429	0.012
Daytime impairment	2.53	0.38	3.23	0.35	1.764	0.190	0.033
Dissatisfaction with sleep	3.36	0.40	4.37	0.37	3.511	0.067	0.063

BIS, Bergen Insomnia Scale; BIS Total, overall severity of insomnia symptoms; M, estimated marginal mean; MBCT, mindfulness-based cognitive therapy; WLC, waitlist control; *p*, value of *p* (two-tailed).^1^Overall insomnia severity based on the composite score of the Bergen Insomnia Scale; ^2^Mean BIS subscale scores = mean number of days per week experiencing the given symptom.*significant at 0.05 level; ES, effect size based on partial eta squared; SE, standard error.

## 4 Discussion

The present study aimed to investigate whether Mindfulness-based cognitive therapy (MBCT) (21) for recurrent depressive disorder alleviated symptoms of insomnia. The majority (83.6%) of the participants screened positive for the insomnia diagnosis as baseline, as assessed through the Bergen Insomnia Scale (BIS). Following 8 weeks of either MBCT or a waiting list control condition, MBCT participants’ overall insomnia symptom severity improved, relative to controls. In terms of specific insomnia symptoms, MBCT participants improved on prolonged sleep onset latency (SOL) and wake after sleep onset (WASO), compared to control participants.

The finding that MBCT participants reported lower overall symptoms of insomnia following the intervention supported the main hypothesis, that MBCT for recurrent depression would alleviate symptoms of insomnia. Furthermore, the improvements in SOL and WASO suggest that MBCT’s effect on the overall burden of insomnia lied in improvement of these specific insomnia symptoms. The results are in line with a growing line of research suggesting that mindfulness-based interventions enhance sleep (26, 27), and indicate that MBCT improves symptoms of insomnia among recurrently depressed individuals.

The findings that MBCT improved participants’ insomnia symptoms are partly in line with Britton and colleagues’ 2012 study, who found that MBCT had beneficial effects on several sleep parameters (35), including total wake time. However, contrary to the present study, Britton’s 2012 study found no benefits of MBCT on prolonged sleep onset or nocturnal awakenings specifically. Results of the present study are also inconsistent with Britton and colleagues’ 2010 trial, which suggested that mindfulness training had some negative effects on participants’ sleep, and no benefits over controls in self-reported sleep (36). The inconsistent findings may lie in methodological disparities between Britton and colleagues’ and the present study. For example, both studies by Britton relied on sleep diaries and polysomnographic assessments, whereas the present study relied on a questionnaire designed to address symptoms typical for insomnia. While polysomnography is often considered the “gold standard” for assessing sleep, insomnia is yet a subjective disorder based on the patients’ perception of their sleep (11). Furthermore, the present study relied on a considerably larger sample size compared to both studies by Britton and colleagues, which may have provided more power to the analyses (50). Nevertheless, the inconsistent results may indicate that additional research on MBCT’s effects on insomnia is needed to fully understand how MBCT affects insomnia.

The non-significant differences in daytime impairment and sleep-related dissatisfaction indicate that although MBCT significantly improved several sleep parameters, the participants did not necessarily perceive their sleep as less problematic following the intervention. Given that the majority of the sample screened positive for the insomnia diagnosis at baseline, and that insomnia seems to predict depressive relapse and recurrence (15, 16), these non-significant findings imply that additional, sleep-specific interventions (i.e., sleep-specific cognitive or behavioral techniques) may be necessary to further improve sleep among recurrently depressed individuals undergoing MBCT. In support of this, an RCT by Ong and colleagues (51) found that mindfulness-based stress reduction, of which MBCT is based (21), was more effective in improving insomnia when combined with sleep-specific treatment components (i.e., from CBT-I). Another possible explanation of the non-significant changes in symptoms among MBCT completers may be that not all participants qualified for insomnia at baseline. This implies that potential benefits of MBCT may have been underestimated to some extent, as one could not expect improvements of insomnia symptoms among insomnia-free participants. Also, it should be noted that the BIS is not validated for investigating single BIS items (e.g., daytime impairment) separately. For future studies, we will therefore recommend including validated tools (e.g., daytime sleepiness scales) to further address how MBCT potentially affects daytime functioning and sleep-related dissatisfaction among insomnia-afflicted individuals.

As the current study was not primarily designed to address MBCT’s effects on insomnia, potential chance mechanisms underlying MBCT’s effects on insomnia were not investigated. Thus, it remains unclear how MBCT was related to the observed improvements of insomnia symptoms. One possibility is that improvements within the emotional, cognitive, and self-relational domains previously shown to improve following MBCT (6, 52) helped MBCT participants overcoming dysfunctional psychological processes assumed to underlie chronic insomnia. For example, and as mentioned previously, it is evident that individuals with insomnia tend to get caught into ruminative thinking when unable to sleep (i.e., repetitively thinking about one’s sleep problem and potential consequences this may have the following day) (32, 53). As ruminative thinking has been linked to increased cognitive and physiological arousal (33), which may increase wakefulness during the pre-sleep period (32), it is likely that reduction in rumination following the MBCT course improved participants’ sleep. Although rumination data were available (6), the sample- and effect sizes were judged too small to include rumination as a potential mediator of the insomnia improvements [see, for example, Qin, 2023; (54)]. In future studies, we will therefore recommend replication using a larger sample size to investigate potential mediators of MBCT’s effects on insomnia.

Furthermore, the finding that 83.6% of the participants screened positive for the insomnia diagnosis at baseline supported the assumption that insomnia would be highly prevalent among recurrently depressed individuals. It is noteworthy that the insomnia rate in the present study exceeded the insomnia rates reported in several epidemiological studies (41, 55), particularly given that participants of the current study were in remission from depression, rather than actively depressed. This finding suggests that recurrently depressed individuals may represent a depression subgroup who are particularly susceptible for developing insomnia. However, as the present findings relied on a small sample size relative to epidemiological studies and the diagnosis of insomnia relied on screening solely, the results should be considered as preliminary and potentially replicated in larger samples with more accurate diagnostic methods.

### 4.1 Strengths and limitations

A strength of the present trial is that it is among few controlled studies to have examined the effects of MBCT on sleep in a sample consisting of individuals diagnosed with recurrent depressive disorder. To our knowledge, the current study is also among the largest RCTs on the effects of MBCT for recurrent depression on insomnia symptoms conducted to date. Results may increase insight in how sleep should be targeted among recurrently depressed individuals.

The present study also had several limitations. First, it should be noted that the BIS does not differentiate between clinical insomnia and other sleep disorders which may yield similar symptoms (i.e., delayed sleep–wake phase disorder, which may easily be confused with prolonged sleep onset due to insomnia, or non-restorative sleep caused by obstructive sleep apnea (11)). For future research, it may therefore be beneficial to include clinical interviews and additional assessment tools to better differentiate between insomnia and other potential sleep disorders. Furthermore, the present trial did not include any exact sleep time estimates. Thus, although MBCT participants improved on several sleep measures relative to controls, it remains unclear whether this change was associated with improvements in, for instance, participants’ sleep duration. Nevertheless, as the diagnostic criteria for insomnia rely solely on subjectively experienced symptoms (1, 11), we argue that subjective measures were appropriate for the present study. Also, it should be noted that as the current study was not designed to address MBCT’s effects on insomnia specifically, the sample size calculations were based on the Beck’s Depression Inventory. Also, as the study was secondary to a larger randomized controlled trial and seven participants did have any no sleep data available at follow-up, no intention to treat analyses were performed. However, when intention to treat analyses were performed in the original trial, the effects of MBCT on the change mechanisms of investigation among completers were either attuned or became non-significant (6). Also, and as reported in the initial publication of the original trial, seven of the non-completers had experienced more than 10 previous depressive episodes, meaning that the program, for example, may have been too comprehensive for these participants (6). Accordingly, the results of the current study should be considered as valid only among completers. Furthermore, it should be noted that the study did not include an active control group. Consequently, it remains unclear whether the observed insomnia improvements could be explained by non-specific treatment factors or placebo effects. Also, and although the study to date is among the largest conducted on potential effects of mindfulness-based interventions on sleep, the sample size was yet relatively small. Finally, the education level in the current sample was generally higher than in the general population. It is therefore uncertain whether the results are generalizable to individuals with lower education levels.

## 5 Conclusion

In conclusion, the rate of participants screening positive for the insomnia diagnosis was high (83.6%) at baseline. Following the eight weeks intervention and when adjusting for baseline insomnia levels, MBCT participants screened lower on the overall severity of insomnia symptoms, as well as on prolonged sleep onset and nocturnal awakenings, compared to waitlist control participants. The results imply that insomnia is highly prevalent in recurrently depressed individuals, and that MBCT may have several beneficial effects on insomnia. However, additional sleep-enhancing interventions may be necessary to further improve sleep among recurrently depressed individuals treated with MBCT.

## Statement of significance

Recurrent depressive disorder is a highly prevalent and often disabling disorder. Research suggests that insomnia increases the risk of relapse and recurrence of depression. Mindfulness-based cognitive therapy (MBCT) is currently the recommended first-line option for recurrent depression, according to the UK National Institute of Health and Clinical Excellence; however, few controlled trials have previously investigated whether MBCT affects insomnia. The present secondary study demonstrated that MBCT improved several insomnia parameters among remitted adult patients with at least three prior episodes of depression. To our knowledge, this is the largest controlled studies on the effects of MBCT on insomnia symptoms conducted to date. Results may enhance the understanding of how insomnia is manifested among recurrently depressed individuals specifically, as well as of how MBCT affects insomnia.

## Data availability statement

The original contributions presented in the study are included in the article/supplementary materials, further inquiries can be directed to the corresponding author.

## Ethics statement

The study was carried out in accordance with the recommendations and approval of the Regional Committee for Medical Research Ethics of Southeast Norway, University of Oslo, Norway, study number 2016/388. All subjects were informed about the study and gave a written informed consent in accordance with the Declaration of Helsinki. All participants provided written consent participate and were told they could withdraw from the study at any time.

## Author contributions

ES and LS conceived and designed the trial. LE wrote the manuscript. ES, LS, LE, and colleges at the Department of Clinical Psychology and the Department of Biological and Medical Psychology at the University of Bergen, Norway, acquired the data. All authors contributed to the conception and hypotheses of the current study, contributed substantially to the statistical analyses and interpretation of data, read and revised the final manuscript, and approved the final version of the manuscript.
